# Latex biomembrane: A new method to coat the open cavity in tympanomastoidectomies

**DOI:** 10.1016/S1808-8694(15)30076-8

**Published:** 2015-10-19

**Authors:** Luiz Carlos Alves de Sousa, Marcelo Ribeiro de Toledo Piza, Joaquim Coutinho-Netto, Danielle

**Affiliations:** a PhD in Neurosurgery - Department of Surgery - Medical School of Ribeirão Preto - University of São Paulo, Professor of Otorhinolaryngology - Medical School of Ribeirão Preto - UNAERP. CEO of the Paparella Association of Otorhinolaryngology; b M.S. in Otorhinolaryngology - Department of Otorhinolaryngology and Ophthalmology - Medical School of Ribeirão Preto - University of São Paulo; Former Fellow of the International Hearing Foundation, University of Minnesota, EUA., Attending Physician - Paparella Association of ENT; c Associate Professor - Department of Biochemistry and Immunology - Medical School of Ribeirão Preto, University of São Paulo; d 3rd Year Intern in ENT - Paparella Association of Otorhinolaryngology. Sociedade Portuguesa de Beneficência Hospital of Ribeirão Preto; e 2nd Year Intern in ENT - Paparella Association of Otorhinolaryngology. Sociedade Portuguesa de Beneficência Hospital of Ribeirão Preto

**Keywords:** wound healing, cholesteatoma, otitis media, otologic surgical procedures

## Abstract

The new cavity created after an open cavity tympanomastoidectomy (OCTM) is filled with an antibiotic impregnated cotton pack (cotton tape, umbilical tape, gauze). The removal of this pack usually causes some bleeding and discomfort for the patient. We propose the use of a latex biomembrane to cover the cavity, which will act as an interface between the raw bone surface and the packing. **Study design:** clinical prospective. **Aim:** To study the performance of the latex biomembrane as an interface between the raw bone surface and the pack, and to analyze its role in cavity epithelization. **Material and Methods:** 64 ears of patients submitted to OCTM were studied. The biomembrane was used in the packing of 54 ears and in the 10 remaining ears the regular cotton tape packing was used. **Results:** In the majority of the cases where the biomembrane was used the packing was removed much easier with no bleeding or pain for the patient and also showed an earlier cavity epithelization. **Conclusion:** The use of the latex biomembrane has proven to be an effective method to cover the mastoid cavity facilitating epithelization and removal of mastoid cavity packing.

## INTRODUCTION

Otitis media (OM) is defined as an inflammatory process, infectious or not, focal or generalized, affecting the auditory cleft^1^. It is one of the most common infectious diseases, bearing relevant consequences for public heath. Its inflammatory process may cause functional hearing loss and may bring about severe complications^2^.

The middle ear inflammatory processes classification proposed by Bluestone & Kenna, in 1988, divides otitis media in suppurative and non-suppurative; acute or chronic and serous or secretory^3^.

Chronic otitis media (COM) is defined as a chronic inflammatory process, located in the auditory cleft, associated or not to tympanic membrane perforation and otorrhea. The alterations associated with this pathology may be summarized in tympanic membrane perforation, ossicles alterations, granulation tissue, cholesteatoma, cholesterol granuloma and tympanosclerosis1.

COM is classified as chronic cholesteatomatous otitis media (CCOM) and chronic non-cholesteatomatous otitis media (CNCOM). Tympanomastoidectomy is the treatment of choice for this disorder, and it may be further divided in canal wall up and canal wall down tympanomastoidectomy, the latter is usually preferred for the treatment of CCOM and sometimes for the treatment of CNCOM because of granulation tissue recurrence^4^.

In order to guarantee surgical success in canal wall down tympanomastoidectomies, it is proposed to open the ear vestibule, thus allowing for a broader access to the surgical cavity, through a broad meatoplasty, which will allow for cavity ventilation; easy visualization and cleaning^5^, ^6^, ^7^.

The newly created cavity by the canal wall down tympanomastoidectomy is filled up with cotton tissue (gauze, threads or cardiac tape), moist in antibiotic ointment. This packing is removed in two weeks, period during which the patient should use systemic antibiotic therapy^4^. This cavity filling method with cotton tissue has been used by us for many years now. In the beginning we used shoe laces made of cotton, but recently we have systematically used cardiac tape. The major problem related to the cardiac tape, or similar agents, happened during its removal, because most of the times the cotton would adhere to the open wound border or to the cavity surface.

Thus, we propose the use of a new biosynthetic material, a natural latex biomembrane to completely coat the cavity surface, serving as a biocompatible interface between the wound area in the neocavity bone and the cotton tissue used in the cavity packing. This biocompatible material is harvested from the rubber tree Hevea brasiliensis, and has been investigated in the department of Biochemistry of the Medical School of Ribeirão Preto - USP since 1994. After harvest, the latex undergoes centrifuging aiming at reducing the amount of proteins naturally present in it; many of them are responsible for allergic reactions. Following that, we add a 4% solution of sulfur and 2% of polyvinylmethylether resin with the goal of providing the final product with elasticity and strength. The homogeneous solution thus formed is placed on Petri dishes, made of glass or acrylic, previously cleaned and dried, where the latex compound is spread until a thin layer is formed covering the dish surface. This process is developed on a flat and leveled table in order to avoid roughness in the liquid that coats the dish. Following that, the dishes are taken to the balanced oven at a temperature of 60 degrees Celsius for a period of 20 minutes and, later, are sterilized in ethylene oxide. Thus, a sterile membrane is formed, which can be used in different situations.

The latex biomembrane has a structure that is very similar to cell membranes. It is chemically produced by the induction of endogenous poly-isoprene polymerization in the lactea acquous emulsion by evaporation at low temperatures, where we can preserve the native proteinase shape, allowing for a reorganization of phospholipids and protein contents in the latex. The special care taken during the polymerization makes the membrane acquire a particular micro-architecture, with a natural surface, which allows cell adherence and stimulates different cell types, especially the polymorphonuclear cells involved in wound healing.

**Figures 1a and 1b:**
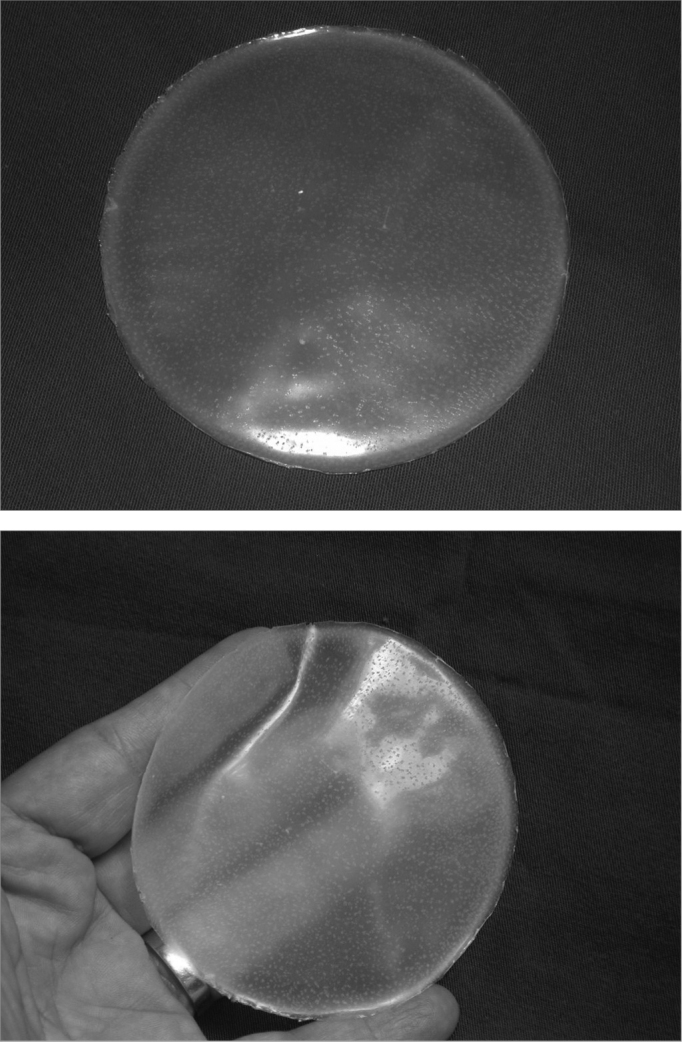
Latex membrane macroscopic aspect.

This biomembrane with such characteristics, may be manufactured in a laminar fashion (being used as a dressing) (Figures 1a and 1b) or in many other formats, such as prosthesis, prepared from special molds^8^, ^9^. Mrué showed that this material is an important healing process inducer in damaged esophageal walls in experimental dogs; and also neoangiogenesis, epithelization (pseudostratified epithelium), submucosal glandular neoformation and muscle fibers10. The biomembrane has been successfully used in a study to treat chronic venous ulcers, in which there was clear evidence of granulation stimulation^11^. Oliveira et al. described the use of this material in myringoplasties^12^.

## OBJECTIVES

The objectives of the present study is to assess the latex biomembrane performance as a biocompatible interface between the bony open wound surface of the newly created cavity and the cotton tissue used for its packing, and to analyze its role in the healing process (epithelization) of the neocavity.

## MATERIALS AND METHODS

The patients selected for the present investigation were followed at the Department of Otorhinolaryngology of the Paparella Association - Santa Lydia Hospital, in Ribeirão Preto. The study was submitted to and approved by the Ethics Committee of the Santa Lydia Hospital - Ribeirão Preto (document # 14/2002) and all patients authorized the use of the material being studied, the storage of data and its use in research.

We analyzed 64 ears of patients from both genders, with ages ranging between 8 and 78 years, who underwent canal wall down tympanomastoidectomies with meatoplasty to treat cholesteatomatous COM or because of granulation tissue recurrence, from January of 2002 to June of 2004. In 54 of the 64 ears operated, after mastoidectomy with a satisfactory lowering of the posterior canal wall down to the level of the facial nerve wall and the making of the “microbox”, we covered the neocavity with the latex membrane and did some relief incisions when necessary in order to facilitate its placement covering the entire neocavity surface. Extreme care was taken in order to avoid leaving areas of bone exposure that could have direct contact with the cardiac tape filled with antibiotic ointment, which was carefully placed over the latex biomembrane, completely covering the neocavity. In the other 10 ears we operated - our control group, we performed the classic packing, in other words, we used only the cardiac tape filled with antibiotic ointment without the latex biomembrane interface.

After two weeks of post-operative we removed the cardiac tape from the 10 ears of the control group. In the 54 ears in which the new method was used, we removed the cardiac tape and, following that, the latex membrane after two weeks of postoperative. In both methods, (64 ears), after removing the packing, we filled up the neocavity with an ointment based on oxytetracyclin and hydrocortisone, to be removed 15 days afterwards (30th postoperative day). After removing this ointment, the patient is instructed as not to let water into the cavity.

**Chart 1:**
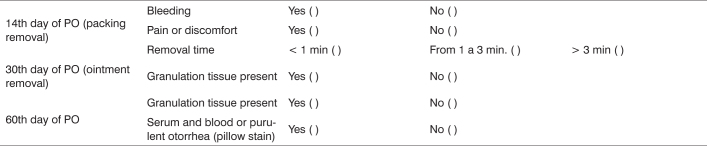
Post-operative assessment guide.

The neocavity was assessed on the 14th, 30th and 60th days of postoperative. The complications of packing removal (bleeding, and pain or discomfort), the time spent in its removal and the healing status of the neocavity were analyzed by means of a protocol that gathered some criteria used for this end (Chart 1).

Assessing the healing status of the neocavity was always carried out by the same examiner. This analysis was held without the examiner knowing whether or not the latex biomembrane was used.

## RESULTS

We analyzed 54 ears of patients with CCOM submitted to canal wall down tympanomastoidectomy and meatoplasty for the placement of latex biomembrane to coat the neocavity. These 54 ears were compared to 10 ears from the control group, in whom only the cardiac tape with antibiotic ointment had been used in order to pack the neocavity, without the latex biomembrane interface.

**Figure 2:**
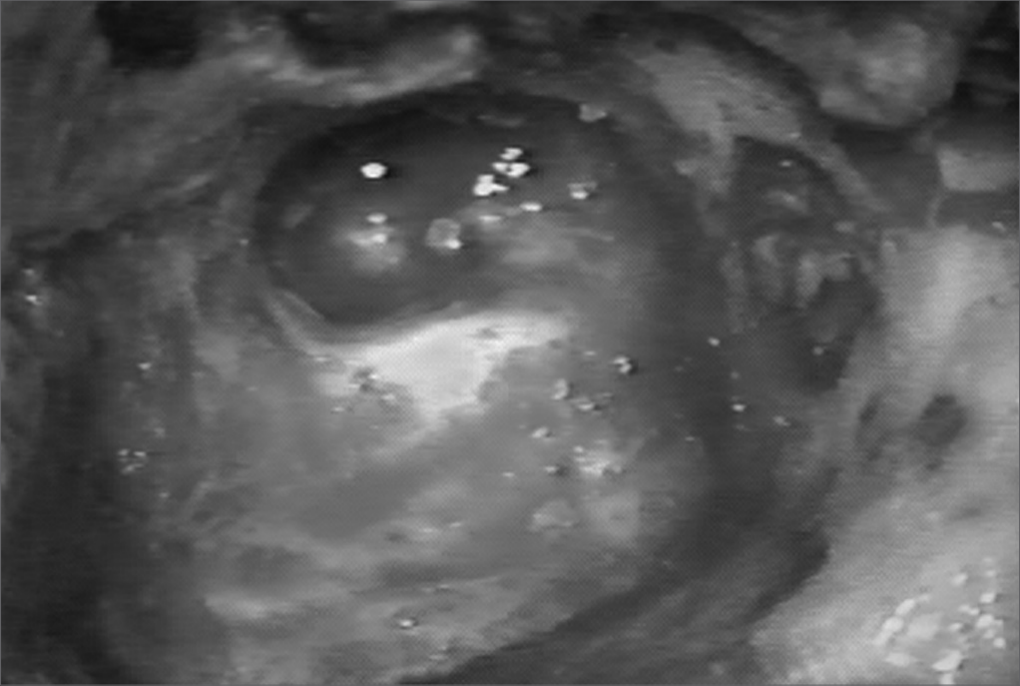
Neocavity aspect at the end of the tympanomastoidectomy.

**Figure 3:**
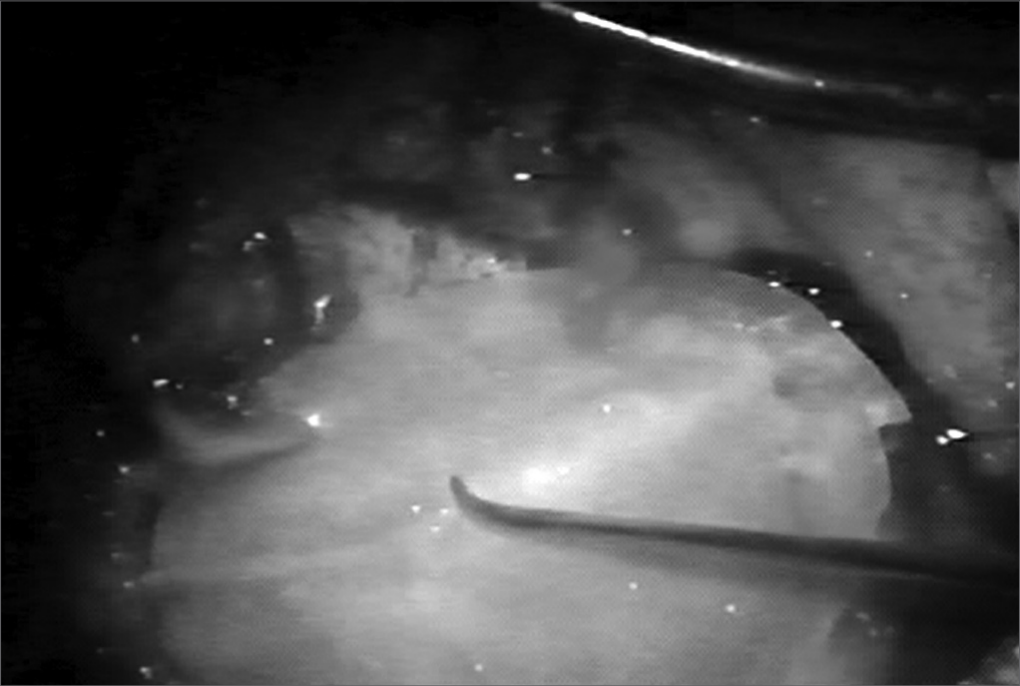
Latex biomembrane being placed on the neocavity.

**Figure 4:**
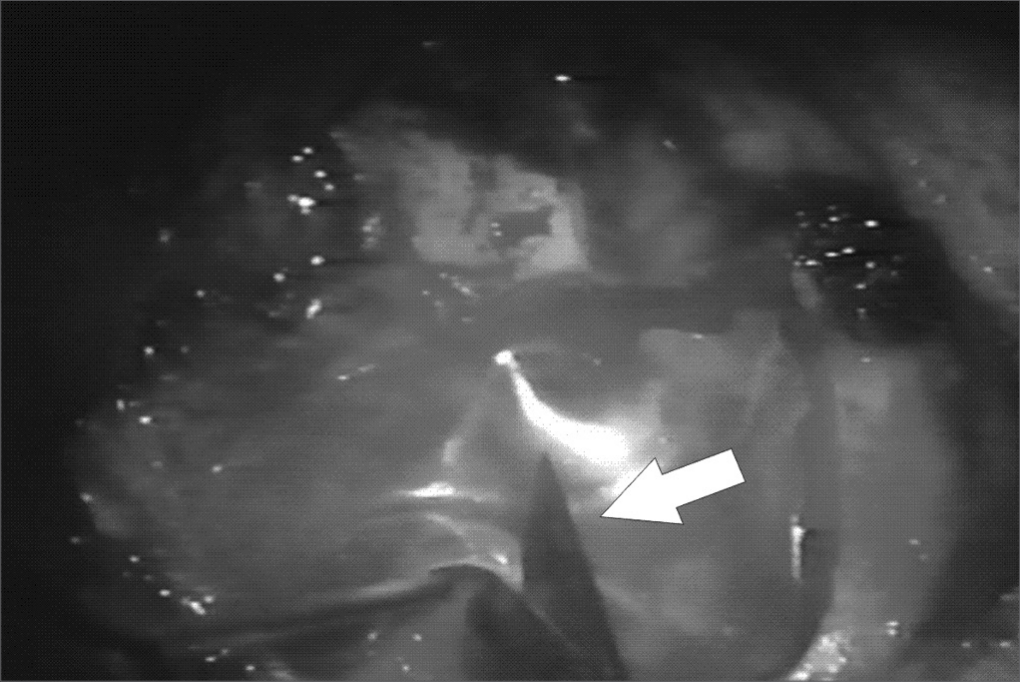
Relief incision in the biomembrane in order to better accommodate it on the cavity.

**Figure 5:**
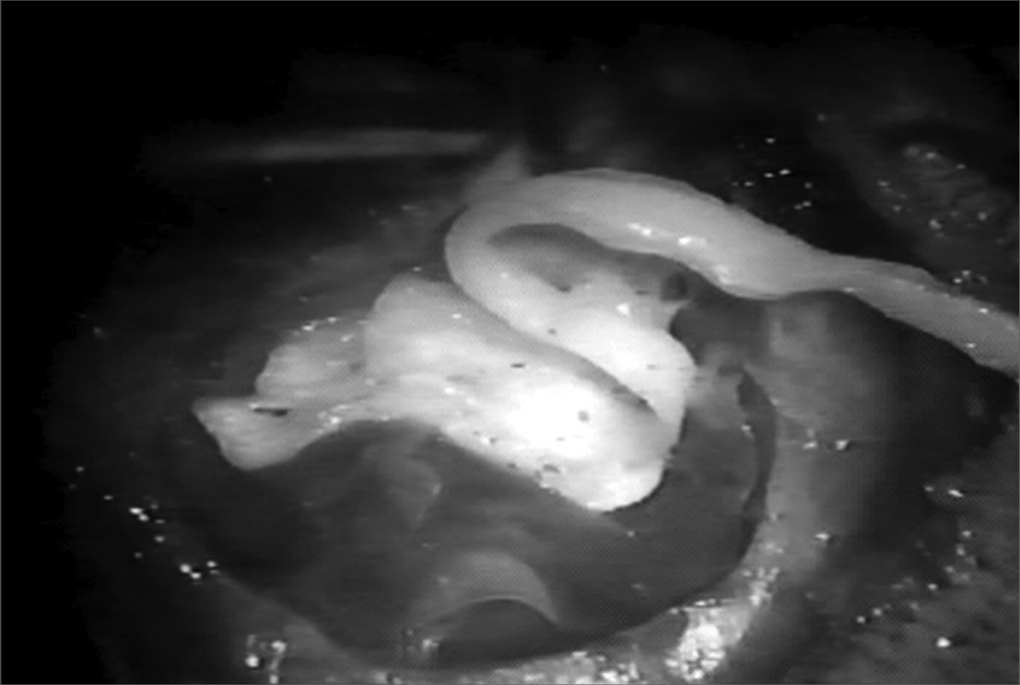
Placing the cardiac tape on the biomembrane.

The neocavity aspect was analyzed at three distinctive moments: at the time of packing removal - on the 14th, the 30th and the 60th day of postoperative (Figures 2, 3, 4 and 5).

At the time of packing removal from the 54 ears in which the latex biomembrane was used, 3 ears (5.5%) presented bleeding at the meatoplasty border and 52 (94.5%) did not present any bleeding. Packing removal caused pain and discomfort in only 3 of these ears that bled. In the 10 ears of the control group, all of them (100%) showed some degree of bleeding, of higher or lower proportion and, sometimes, it was necessary to use chemical cautery procedures in order to control bleeding. All the patients in the control group reported pain or discomfort at the time of packing removal.

In all the 54 ears in which we used the latex biomembrane, it took the most of 1 minute to remove the packing. Of the 10 ears in the control group, in two of them the time taken to remove the packing was less than 1 minute; in seven it took between 1 and 3 minutes; and in one of them, this time was longer than 3 minutes.

After removing the ointment of the neocavity in the 30th day of postop granulation tissue was seen in 8 (14.8%) of the 54 ears in which we used the latex biomembrane. In the 10 ears of the control group we observed the presence of granulation tissue in 6 of them (60%).

60 days after surgery, 5 (9.3 %) of the 54 ears still presented areas of granulation tissue on the neocavity surface. In the control group we observed granulation tissue in 2 (20%) of the 10 ears operated upon. The complain of serous and bloody or purulent otorrhea at this time of post-operative, happened in 6 (11.1%) of the 54 ears in which the latex biomembrane was used, and in 3 (30%) of the 10 ears in the control group.

**Figure 6:**
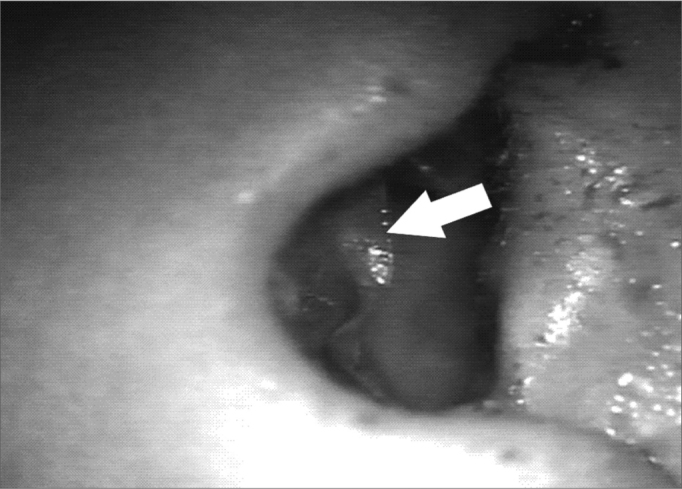
Latex biomembrane being removed on the 14th day of postoperative.

**Figure 7:**
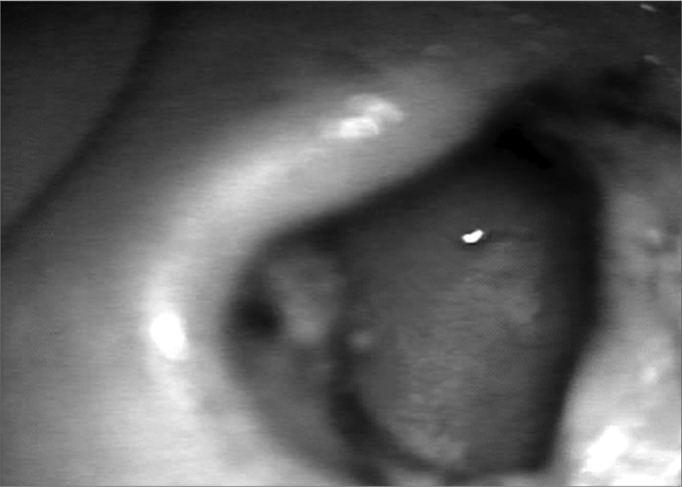
Neocavity surface aspect showing a thin fibrin layer after removing the latex biomembrane on the 14th P.O. day.

**Figure 8:**
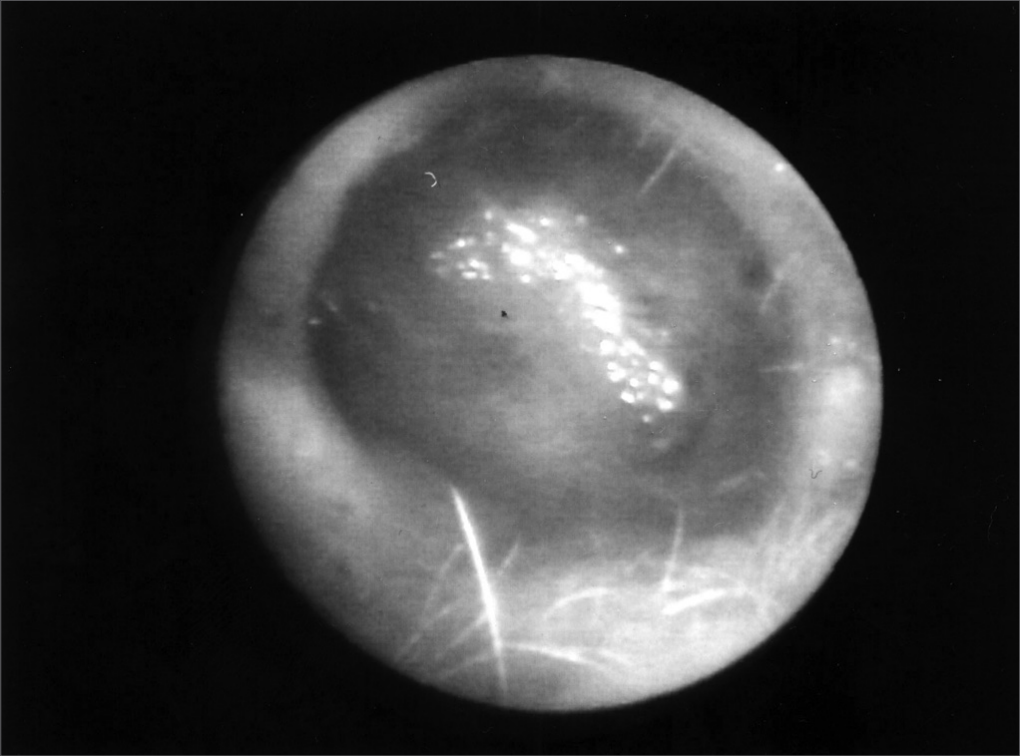
Neocavity totally epithelized on the 60th day of P.O.

In the first analysis (14th day of postoperative) we observed a thin layer of fibrin on the neocavity surface, which was seen after the removal of the latex biomembrane (Figures 6 and 7). In the control group, this surface was bloody and often times already had some granulation tissue after removing the cardiac tape. On the 30th and 60th days of postoperative it was clear that we had a better epithelization of the neocavity in the patients in whom we used the biomembrane (Figure 8), when compared to the control group. We observed that the patients in whom the biocompatible material was used had an earlier neocavity epithelization, when compared to the ears in which only the cardiac tape with ointment filled the neocavity.

## DISCUSSION

For more than two decades we have faced the difficulty in finding the best way to fill the newly-created canal wall down tympanomastoidectomy cavities. We have used Gelfoam®, gauze, shoe laces and, more recently, the antibiotic ointment filled cardiac tape. Under such situations it was not uncommon the need for a frequent cleaning of the neocavity for the removal of granulation tissue and debris, which sometimes peaked with the need to coat it with free skin grafts taken from the forearm, method proposed by Ollier-Thiersch^13^, ^14^.

It must be stressed that the packing removal at the 14th day of postoperative, is already a procedure that causes pain and discomfort for the patient and for the physician, and it is more time consuming because of the packing material adherence to granulation tissue areas within the neocavity. Often times, this removal caused diffuse bleeding which required cauterization of the neocavity surface.

Because of these difficulties in removing the packing, we deem it useful to coat the whole neocavity surface with an interface made of a biocompatible material - the latex biomembrane, which is placed between the cotton shoelace (cardiac tape) and the cavity surface represented by the exposed bone, skin flaps and gelfoam which covers the graft in the “microbox”.

As we remove the shoelace and, after that, the latex from the cavity of the 54 ears in which we used the new method, the biomembrane easily slid over a thin fibrin layer, which served as a cradle for the growth of coating epithelium. During such removal we did not observe any bleeding or patient discomfort, and the same did not happen in the control group cases. In these cases, it is very likely that the bleeding and the granulation tissue were responsible for the delay in neocavity epithelization. The fact that we did not traumatize the neocavity surface when we removed the cardiac tape - which did not adhere to the bone surface - was due to the protection offered by the biomembrane, it reduces bleeding and repair tissue formation, thus creating a more suitable surface to bear the epithelization process in this newly created cavity.

We decided to use the latex biomembrane to serve as an interface that will protect the neocavity surface and, it is very likely that, will precipitate the epithelization process thanks to its neoangiogenesis and healing properties. Many papers have been published regarding its applicability, especially for the treatment of leg ulcers^8^, ^9^, ^11^.

It is worth mentioning that the packing removal (cardiac tape and biomembrane) and the full access to the neocavity can only be possible thanks to the opening created during meatoplasty, thus the importance of having this procedure done in the meatus. We believe that, when we create an open cavity, it is paramount to make a meatus of the proper size, which allows us to approach the cavity in all its extension.

The difficulties the otorhinolaryngologist faces using only the cotton tissue to coat the neocavity, are brought about by its adherence to the neocavity surface are patient discomfort and pain; and the greater need for time to control these complications happened in a much lower rate in the group that received the latex membrane.

The neocavity epithelization happened successfully in most of the patients who received the new method. This may be explained by the fact that the biomaterial used stimulates neovascularization and organized tissue growth in different body organs and tissues, and it is an innocuous material that is not rejected by the body.

We believe that the use of latex biomembrane in canal wall down tympanomastoidectomies be but a detail of the surgical technique that may enhance the work of the surgeon in the care given to the neocavity in the post-operative, besides probably promoting a faster epithelization process in this cavity.

## CONCLUSION

The latex biomembrane in canal wall down tympanomastoidectomies has proven to be of great efficiency as a biocompatible interface with neoangiogenesis and healing capacities between the open bone wound and the cotton tissue used for cavity packing, thus facilitating packing removal and cavity epithelization.

## References

[1] Costa SS, Paparella MM, Cruz OLM, Costa S, Cruz O, Oliveira J (1994). Otorrinolaringologia. Princípios e Prática.

[2] Meyerhoff WL, Kim CS, Paparella MM (1978). Pathology of chronic otitis media. Ann Otol Rhinol Laryngol.

[3] Bluestone CD, Kenna MA (1988). Workshop on chronic suppurative otitis media: etiology and management - Proceedings. Ann Otol Rhinol Laryngol.

[4] Cruz OLM, Costa SS, Cruz OLM, Costa SS (2000). Otologia. Clínica e Cirúrgica.

[5] Sousa LCA, Piza MRT, Costa SS, Cruz OLM, Costa SSd (2000). Otologia Clínica e Cirúrgica.

[6] Sousa LCA (1996). Meatoplasty. Operative Techniques in Otolaryngology-Head Neck Surg.

[7] Sousa LCA, Piza MRT, Andrade MJ, Jaeger WL, Nascimento JMN (1996). Meatoplastia: Indicações, Vantagens e limitações. Rev Bras Otorrinolaringol.

[8] Frade MAC, Cursi IB, Andrade FF, J C-N, Foss NT (2002). Stimulation of diabetic wound healing by natural latex biomembrane (NLB). Annales de dermatologie et de vénéréologie (20th World Congress of Dermatology).

[9] Frade MAC, Cursi IB, J C-N, Foss NT (2002). Induction of leg wound healing by natural latex biomembrane (NLB). Annales de dermatologie et de vénéréologie (20th World Congress of Dermatology).

[11] Frade MAC, Valverde RV, J C-N, Foss NT (2001). Chronic phlebopathic cutaneous ulcer: a therapeutic proposal. Int J Dermatol.

[12] Oliveira J, Hyppolito MA, Coutinho-Netto J, Mrué F. (2003). Miringoplastia com a utilização de um novo material biossintético. Rev Bras Otorrinolaringol.

[13] Ollier LXEL (1872). Greffes cutanées ou autoplastiques. Bulletin de l’Académie de médecine.

[14] Thiersch K (1874). Ueber die feineren anatomischen Veränderungen bei Aufheilung von Haut auf Granulationen.

